# Estimated pre-morbid IQ effects on cognitive and functional outcomes in Alzheimer disease: a longitudinal study in a treated cohort

**DOI:** 10.1186/1471-244X-8-27

**Published:** 2008-04-21

**Authors:** John M Starr, Jane Lonie

**Affiliations:** 1 Geriatric Medicine unit, University of Edinburgh, Craigleith Road, Edinburgh, UK; 2 Lothian Memory Treatment Service, NHS Lothian, Royal Edinburgh Hospital, Edinburgh, UK

## Abstract

**Background:**

Cognitive reserve is thought to influence the degree of neuropathology needed for diagnosis of Alzheimer disease (AD). Cognitive reserve can be operationally defined as the hypothesized capacity of the mature adult brain to sustain the effects of disease or injury without manifesting clinical symptoms of AD, but sufficient to cause clinical dementia in an individual possessing less cognitive reserve. Its effect on the subsequent course of AD is less clear. Pre-morbid IQ is a useful measure of cognitive reserve.

**Methods:**

We studied 659 consecutive patients with AD at a tertiary referral memory clinic. Patients were assessed on six cognitive tests at baseline. Activities of Daily Living (ADL) were measured on the Instrumental Activities of Daily Living (IADL) scale and Physical Self-Maintenance Scale (PSMS). The National Adult Reading Test (NART) was used to estimate pre-morbid IQ. Patients were followed up after starting a cholinesterase inhibitor over 78 weeks. Mixed general linear models estimated the effects of NART on cognition and ADL.

**Results:**

Three hundred and fifty-five patients had NART scored with a mean estimated pre-morbid IQ of 104.7 (standard deviation 18.5). NART increased overall cognitive ability by 2.7% for every 10 IQ points (p < .001). There was a trend for an effect on the change in cognition over time (p = .065) with higher NART associated with improvement of cognitive ability over time. After adjusting for age and sex, a 10 point increase in NART was associated with an improvement of 2% in ADL scores, but this effect was explained by NART's influence on contemporaneous cognitive ability.

**Conclusion:**

Our data support the hypothesis that cognitive reserve continues to have a limited influence on cognition after AD has been diagnosed and thus, indirectly, has an impact on ADL.

## Background

Cognitive loss resulting in impairment of activities of daily living (ADL) is central to the diagnosis of Alzheimer disease (AD) [1]. Cholinesterase inhibitors, which are symptomatic treatments, aim to halt and reverse decline in both cognitive abilities and ADL [2]. Evidence from randomised clinical trials suggests that all available cholinesterase inhibitors have similar, moderate beneficial effects on these outcomes [3]. However, not all patients who take cholinesterase inhibitors benefit and there are no reliable predictors of whom is more or less likely to benefit from these drugs. Nevertheless, despite a lack of evidence of particular patient groups that might benefit most, evidence from longitudinal studies prior to the pervasive use of cholinesterase inhibitors is able to inform clinicians which patients might be at greatest risk of decline if left untreated. Review of available studies indicates that, paradoxically, people with AD who have a greater pre-morbid cognitive reserve are those who suffer the most rapid decline after diagnosis, at least in terms of life expectancy [4]. This is in contrast to the observation that increased cognitive reserve protects against the development of AD [4]. It is hypothesised that a greater pathological load is needed to produce the same severity of dementia in those with increased cognitive reserve and that dementia severity, itself, predicts rate of subsequent decline [4]: the course of decline in this paradigm, linear or non-linear, is not yet established. The precise biological substrates that underpin cognitive reserve require further elucidation but can be considered in terms of those associated with neurodevelopment (e.g. white matter integrity [5]) and those associated cognitive ageing (inflammation, oxidative stress and apoptosis) [6]. Such substrates are thought to relate both to 'passive' reserve (i.e. fixed structures) and 'active' reserve (i.e. the functional capacity of the brain to remodel, reconfigure etc) [7].

There is a wide range of putative indices of cognitive reserve [4], chief amongst which is pre-morbid IQ [7]. Although cognitive reserve is thought to have its primary effect on cognitive ability, its impact on ADLs, which may be secondary to its effect on cognition, is also important, especially in the context of dementia [7]. ADL criteria may be superior to brief screening tests in screening for AD in people with low educational attainment [8] suggesting that education, and hence cognitive reserve, may be less closely associated with ADLs than with cognition. Despite a paucity of relevant data, support for this hypothesis comes from a study of nearly 500 people with AD in Ohio where education, but not occupation, influenced rate of cognitive decline, whilst neither education nor occupation affected rate of decline in ADLs [9].

One methodological limitation of using education as an index of cognitive reserve is that, unlike pre-morbid IQ, it typically has limited variance, especially in women. There is evidence in healthy older people that higher actual pre-morbid IQ is associated with better ADLs in old age, even after adjusting for contemporaneous cognitive ability [10]. However, pre-morbid IQ accounts for only around 2.5% of variance in ADL scores [10] compared with around 50% of IQ measured in old age [11]. It is thus unsurprising that any effects of education on ADL in older people are difficult to detect compared to its effect on cognitive test scores. The corollary of this is that investigation into any effect of cognitive reserve in AD is likely to require sensitive measures of cognitive reserve, such as pre-morbid IQ, rather than coarser measures, such as education and occupation, to have adequate power to detect significant effects in all but the largest samples. Unfortunately, AD samples in which pre-morbid IQ is available are rare and researchers have to rely on proxy variables that estimate pre-morbid IQ. But here the question arises as to whether these proxy variables have, themselves, been affected by the disease process.

The National Adult Reading Test (NART) [12] is commonly used to estimate pre-morbid IQ. Using childhood mental ability scores collected for a Scottish national survey at age 11 in 1932 [13] the NART has been validated both in a healthy sample [14] and in people with dementia who form part of the cohort described in this study [15]. NART estimated IQ (NARTIQ), together with age, is a major predictor of Mini-Mental State Examination (MMSE) scores in health [16], but also in dementia across a wide range of severity [17]. However, its influence on other cognitive tests and on ADL is unknown in people with AD. It is unclear, for example, whether any association diminishes as the disease progresses. If this were the case, it might imply that cerebral reserve becomes less important as AD becomes more severe and that any protective effect of cognitive reserve is lost over time. This would provide an explanation for the paradoxical observation that people with greater cognitive reserve decline more quickly once they are diagnosed with AD which otherwise is explained by assuming that mortality is associated with the degree of neuropathology rather than the clinical severity of dementia. In addition, quantifying relationships between NARTIQ and cognitive tests would allow more accurate adjustment for pre-morbid IQ when neuropsychological assessment forms part of the diagnostic process.

## Methods

### Sample

The sample, described in detail elsewhere [17,18], comprised 659 (195 male, 464 females) consecutive patients with probable AD treated at a tertiary referral memory clinic. Diagnosis was made or reviewed according to NINCDS-ADRDA criteria at the clinic and patients were scored on a range of cognitive and functional independence measures at baseline (see below). Choice of cholinesterase inhibitor drug was pragmatic depending on whether twice daily compliance could be assured (rivastigmine given) or only once daily (donepezil given). Patients were treated for 26 weeks and then reassessed on cognitive, ADL, behavioural and carer-rated global measures as described below. A small proportion of patients seen during the initial phase of the clinic were also tested after 12 weeks of treatment. Response was judged at 26 weeks so that once we were happy that the tests could be used repeatedly in our patients we no longer tested at week 12. A multi-disciplinary team comprising two consultant psychiatrists, a consultant physician, a neuropsychologist, two nurses and a pharamacist decided whether the patient had responded to treatment. In general patients were considered as responders if cognitive scores improved or were stable over 26 weeks. Responders were continued on the same drug and underwent further assessments at 52 weeks and 78 weeks after treatment initiation.

### Measures

Cognitive testing comprised the MMSE (score range 0–30) [16], the Hopkins Verbal Learning Test (HVLT) (range 0–72) [19], Verbal Fluency Categorical (VF-Cat), totalling the number of animals named, and Lexical (VF-Lex), totalling 'F', 'A' and 'S', forms [20], and the Paired Associate Learning (PAL) and Delayed Matching to Sample (DMTS) subtests from the Cambridge Neuropsychological Test Automated Battery visual and working memory battery [21]. Pre-morbid IQ was estimated with the NART a test of ability to pronounce 50 irregularly spelt English words [12]. Functional independence was measured on the Instrumental Activities of Daily Living (IADL) scale (range 0–32) [22] and Physical Self-Maintenance Scale (PSMS) (range 0–24) [22] commonly used as outcome measures in AD [23]. Unlike the cognitive tests, higher IADL scores indicate impairment due to greater dependency.

### Statistical analyses

All statistical analyses were performed using the SPSS 14.0 statistical package. After initial data description, we used a general mixed linear models approach similar to that adopted for the analysis of several waves of cognitive data in a study of healthy cognitive ageing that estimated the effects of prior IQ [24]. This approach allows data to be included from participants even when they have not attended all waves. Dummy variables were produced to signify which waves patients had attended so that any effect of attendance on dependent variables – which was likely given that only responders attended at weeks 52 and 78 – could be adjusted for. There were two data types: 1) the response variables, cognitive test scores and ADL scores that were measured at each wave; 2) and fixed explanatory variables including NARTIQ. The six cognitive test scores were treated as repeated measures at each assessment wave for an underlying cognitive trait and between waves as snapshots over a relatively short period. Formally, the model was of the form y = Xβ + Zγ + ε where X is the matrix of fixed effects, Z the matrix of random effects, β the vector of fixed effects parameters, γ the vector of random effects parameters and ε the vector of residual error. Hence the mixed model was set up to test for effects of fixed variables on an underlying cognitive trait derived from variance common to all cognitive tests and stable over time. The model could also test for effects of fixed variables on change in this cognitive trait between waves. Since such effects tested the interaction between fixed variables and wave, statistical power to detect significant effects was less than that to detect effects on the underlying cognitive trait. Similarly the model could test for effects of fixed variables between different cognitive tests. Finally, the model could test for differential effects of fixed variables on change with time between the cognitive tests. Models were adjusted for age, sex and drug given as co-variables.

This design allowed adjustment for the high inter-correlation between cognitive tests and between waves whilst allowing investigation of effects specific for each test or at each wave after adjustment for the general effect. The model adjusts for these inter-correlations by pre-specifying contrast matrices related to the covariance structure of the data. However, such contrast matrices cannot be assumed *a priori *but, instead, the data have to be examined to determine which covariance structure fits best. Goodness of fit is determined using standard information criteria. Once the optimal covariance structure is determined, the effects of fixed variables can then be tested for. Thus, using a restricted maximum likelihood method, we compared different covariance structures for within subject residual errors to determine the optimal covariance structure judged by -2 restricted log likelihood, Akaike Information Criterion (AIC) and Schwarz's Bayesian Information Criterion (BIC) measures for the model with no factors or covariates entered. A heterogeneous autoregressive covariance structure was found to be optimal and this was set for subsequent hypothesis testing of the fixed effects. IADL and PSMS were treated similarly to the cognitive test scores and considered to represent an underlying ADL trait. A first order autoregressive covariance structure was found to be optimal for ADL data. Finally, of note is that for each hypothesis tested the denominator degrees of freedom might have non-integer values since these were derived by a Sattlethwaite approximation.

## Results

### Sample description

Four hundred and thirty-three (65.7%) of the sample were treated with donepezil and 226 (34.3%) with rivastigmine. Three hundred and fifty-five patients had NARTIQ scored with a mean estimated pre-morbid IQ of 104.7 (standard deviation 18.5) and mean age 77.4 years (standard deviation 7.1 years), 70% of whom were women and 19% lived alone. Patients with NARTIQ scores had significantly better scores on all the cognitive tests than those who did not have NARTIQ scores (differences all p < .001 except PAL p = .011 and DMTS p = .008). Mean cognitive test scores for all waves of assessment are shown in Table 1 and mean IADL and PSMS scores in Table 2. Failure to be assessed after 26 weeks treatment, reflecting poor drug tolerance or concordance, was not significantly associated with sex (χ^2 ^= .52, p = .47), living alone (χ^2 ^= 2.55, p = .11), drug given (χ^2 ^= .003, p = .96), age (F = .87, p = .35), NARTIQ (F = 2.30, p = .13), baseline MMSE (F = .63, p = .43), IADL score (F = .30, p = .58) or PSMS score (F = .02, p = .90). Having a week 52 assessment, indicative of positive response to cholinesterase treatment, was not significantly associated with sex (χ^2 ^= .05, p = .83), drug given (χ^2 ^= .00, p = 1.0), age (F = .23, p = .63), NARTIQ (F = 2.61, p = .11), baseline MMSE (F = 1.22, p = .27). There was a trend for benefit for those patients who did not live alone (Fisher's exact test p = .053). Responders had significantly lower (better) baseline IADL scores (mean 14.1 versus 16.1, F = 6.66, p = .010) and PSMS scores (mean 6.8 versus 8.1, F = 12.21, p = .001).

**Table 1:** Mean cognitive test scores at baseline (week 0) and after 12, 26, 52 and 78 weeks of cholinesterase inhibitor treatment for all patients and the sub-sample who had NARTIQ scores.

**Test (week)**	**N **All patients	**Mean score**	**Standard deviation**	**N **Patients with NARTIQ scores	**Mean score**	**Standard deviation**
**MMSE(0)**	626	19.3	5.8	346	20.7	4.9
**HVLT(0)**	601	9.2	4.9	342	10.0	5.0
**VF-Cat(0)**	604	7.8	4.3	344	8.7	4.4
**VF-Lex(0)**	604	22.0	13.1	342	24.8	13.5
**PAL(0)**	461	4.3	1.8	298	4.5	1.8
**DMTS(0)**	447	11.1	3.1	290	11.5	2.8
**MMSE(12)**	143	19.5	5.5	101	20.3	5.2
**HVLT(12)**	84	9.7	5.6	67	10.2	5.7
**VF-Cat(12)**	82	8.5	5.0	66	9.0	5.3
**VF-Lex(12)**	82	24.3	14.9	66	25.7	15.2
**PAL(12)**	70	4.2	1.7	59	4.1	1.7
**DMTS(12)**	71	10.8	2.9	58	11.0	2.9
**MMSE(26)**	259	19.0	5.6	162	19.7	5.5
**HVLT(26)**	250	9.5	4.6	157	9.9	4.9
**VF-Cat(26)**	248	7.6	4.2	158	8.1	4.5
**VF-Lex(26)**	248	23.2	12.5	158	24.8	12.5
**PAL(26)**	170	4.5	1.8	124	4.4	1.7
**DMTS(26)**	183	11.1	3.1	127	11.2	3.0
**MMSE(52)**	96	18.6	5.4	75	19.0	5.3
**HVLT(52)**	87	9.2	5.7	69	9.3	5.8
**VF-Cat(52)**	87	6.9	4.5	68	7.0	4.7
**VF-Lex(52)**	87	23.5	13.9	68	25.0	14.0
**PAL(52)**	76	4.4	1.6	62	4.3	1.6
**DMTS(52)**	75	10.9	2.8	58	10.7	2.8
**MMSE(78)**	43	19.2	5.5	33	19.6	5.7
**HVLT(78)**	38	9.9	6.2	20	10.3	6.3
**VF-Cat(78)**	39	7.6	4.7	31	7.9	5.0
**VF-Lex(78)**	39	25.1	13.6	31	26.9	13.5
**PAL(78)**	29	4.3	1.8	23	4.3	1.8
**DMTS(78)**	33	10.6	3.4	25	10.7	3.4

MMSE – Mini-Mental State Examination; HVLT – Hopkins Verbal Learning Test total score over six trials; V-Cat – Categorical Verbal Fluency; V-Lex – Lexical Verbal Fluency; PAL – Paired Associates Learning from CANTAB battery; DMTS – Delayed Matching to Sample stages from the CANTAB battery.

**Table 2:** Mean IADL and PSMS scores for all patients and the sub-sample who had NARTIQ scores at baseline (week 0) and after 26, 52 and 78 weeks of cholinesterase inhibitor treatment; higher scores indicate greater dependency

**Assessment**	**N **All patients	**Mean**	**Standard deviation**	**N **Patients with NARTIQ	**Mean**	**Standard deviation**
**IADL(0)**	551	15.8	6.5	294	14.7	6.2
**PSMS(0)**	548	7.9	3.1	292	7.5	2.9
**IADL(26)**	238	17.3	5.9	146	16.9	5.9
**PSMS(26)**	237	8.5	3.0	146	8.3	2.8
**IADL(52)**	75	16.5	7.2	59	16.7	7.1
**PSMS(52)**	74	8.1	3.5	58	8.1	3.4
**IADL(78)**	46	18.0	6.1	39	18.0	6.5
**PSMS(78)**	46	8.5	2.7	39	8.4	2.8

### Effects of NARTIQ on cognition

NARTIQ had a significant main effect on the underlying cognitive trait as measured repeatedly by the six cognitive tests over the five waves of assessment (F_1,245.7 _= 77.2, p < .001). NARTIQ also made a significant differential contribution to the six cognitive tests (F_5,157.5 _= 18.68, p < .001) and had a significant effect on the differential change in cognitive test scores over time (F_20,217.5 _= 1.66, p = .043). There was a statistical trend for an effect on the change in the underlying cognitive trait over time (F_4,243.0 _= 2.24, p = .065) with higher NARTIQ being associated with improvement of cognitive ability up to 78 weeks. Age, but not sex or drug given, also had significant effects on cognition. There was a significant main effect (F_1,320.6 _= 7.68, p = .006) and a differential effect between cognitive tests (F_5,228.0 _= 3.16, p = .009), but no effects on change in cognitive test scores over time. The model set mean age at 77.3 years and for every additional 10 years overall cognitive ability was 3.5% lower. Relative to MMSE, age had a less adverse effect on PAL (0.6% per 10 years) but, in order, an increasingly adverse effect on VF-cat (0.1% per 10 years), HVLT (0.1% per 10 years), DMTS (0.3% per 10 years) and VF-lex (0.3% per 10 years). Adjusting for age and NARTIQ, there was no significant change in cognitive ability over time (F_4,254.0 _= 1.85, p = .12), nor in differential change in cognitive ability between cognitive tests over time (F_20,226.0 _= 1.06, p = .39).

**Figure 1:**
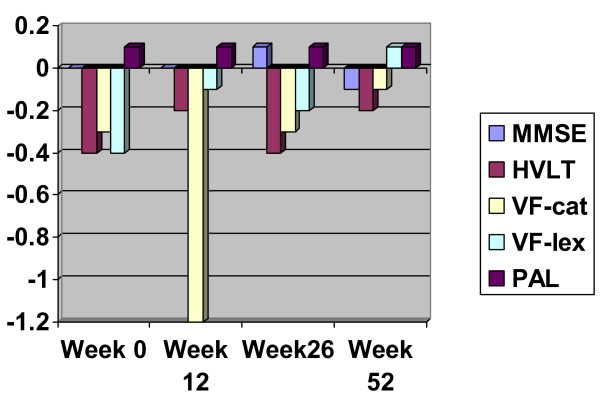
**Percentage change of cognitive test scores relative to DMTS per 10 points increase in NARTIQ**. MMSE – Mini-Mental State Examination; HVLT – Hopkins Verbal Learning Test total score over six trials; V-Cat – Categorical Verbal Fluency; V-Lex – Lexical Verbal Fluency; PAL – Paired Associates Learning from CANTAB battery; DMTS – Delayed Matching to Sample stages from the CANTAB battery.

The main effect of NARTIQ increased overall cognitive ability by 2.7% for every 10 IQ points. Relative to MMSE, NARTIQ had a more positive effect, in increasing order, on VF-cat (0.1% per 10 points), HVLT (0.2% per 10 points), and VF-lex (0.6% per 10 points), but had a lesser effect on DMTS (0.1% per 10 points) and least of all on PAL (0.3% per 10 points). Figure 1 shows the differential effect of NARTIQ on cognitive tests over time. MMSE, HVLT, PAL and DMTS are essentially unaffected by NARTIQ, but for those patients with higher NARTIQ scores, VF-cat shows a dip at week 12 whilst VF-lex shows a steady improvement.

### Effects of NARTIQ on ADL

NARTIQ had a significant main effect on ADL as measured repeatedly by IADL and PSMS at baseline, week 26, week 52 and week 78 of treatment (F_1,114.6 _= 5.69, p = .019). NARTIQ also had a significant effect on change in ADL over time (F_3,62.4 _= 4.13, p = .010). NARTIQ had no significant differential effect on IADL compared with PSMS (p = .27) or on differential change over time (p = .11). Age and sex, but not drug given or living alone, also had significant effects on ADL. In a model that included NARTIQ, age and sex, women had significantly worse ADL scores over all waves compared with men (mean 12.7 for women, 11.5 for men, F_1,124.6 _= 4.03, p = .047), with significantly worse IADL scores (mean 17.7 for women, 15.4 for men on IADL and 8.6 for women and 8.7 for men on PSMS, F_1,148.5 _= 15.09, p < .001) after adjusting for other effects; there were no significant effects of sex on change in ADL over time. There was a statistical trend for increasing age to be associated with worse ADL (F_1,117.8 _= 3.09, p = .082), but no significant effect over time (p = .34). Older people scored differentially worse on IADL compared with PSMS (F_1,143.0 _= 4.82, p = .030) with a trend for this to be attenuated over time (F_3,56.4 _= 2.63, p = .059). After adjusting for age, sex and NARTIQ, there was a significant deterioration in ADL scores over time (F_3,44.4 _= 4.60, p = .007), more markedly for IADL rather than PSMS (F_3,58.2 _= 3.90, p = .013).

After adjusting for age and sex, a 10 point increase in NARTIQ was associated with an overall improvement of 2% in ADL scores. In addition, for every 10 extra NARTIQ points, there was an improvement of 2% in ADL scores between baseline and week 78. To check whether the effect of NARTIQ was explained by current rather than pre-morbid cognitive ability, baseline MMSE score was introduced as an additional independent covariable into the model. All of the significant effects of NARTIQ previously found became non-significant indicating that effects of current cognitive ability on ADL in AD far outweigh those of pre-morbid IQ.

## Discussion

Pre-morbid IQ continues to influence a range of cognitive tests after AD diagnosis. The effect size is modest, around 2.7% for every 10 IQ points, compared with its effect in non-demented older people [14]. Unsurprisingly, the effect is greater for verbal compared with non-verbal tests. There was a trend for higher pre-morbid IQ to improve performance on cognitive scores over time for most tests relative to lower pre-morbid IQ, with higher pre-morbid IQ being particularly associated with relative improvement in lexical verbal fluency, though this may reflect a relative lack of novelty on repeated testing. Being older also has a detrimental effect on cognitive scores, even in the presence of dementia, but has no effect on change in cognitive ability over time. As expected, given the selection of responders to continue treatment, there was no significant effect of treatment on cognitive change over time. However, despite this cognitive stability, ADL scores deteriorated, especially instrumental activities of daily living. This may reflect a greater sensitivity of IADL to change compared with PSMS. The effect size of pre-morbid IQ on ADL scores was similar to that of its effect on cognition. The effect size was similar to that seen cross-sectionally [10]. Again, those with higher pre-morbid IQs seemed to be relatively protected from any deterioration in ADL over time. However, once the effect of contemporaneous cognitive ability was adjusted for, pre-morbid IQ no longer exerted a significant effect on ADL. Thus the effects of pre-morbid IQ on ADL appear to be mediated via its effects on current cognitive ability in AD. In this sample, younger men scored significantly better on instrumental activities of daily living, perhaps reflecting persisting social roles. This effect was distinct from any effect of living alone, suggesting that it could not be explained purely by the likelihood of younger men having wives who were still alive. It may, on the other hand, be a cohort effect of lower pre-morbid prevalence of performing household tasks like cooking and laundry in men who married before the end of the Second World War.

The effect of pre-morbid IQ on cognition in this longitudinal study was considerably less than its effect on MMSE on a cross-sectional basis [17]. This is likely to reflect the superior design of longitudinal studies that can adjust for within-subject effects that might otherwise be attributed to a stable trait such as pre-morbid IQ. Despite the moderate effect size, its presence supports the persisting effect of cognitive reserve on the absolute level of cognition, and to a lesser extent the rate of cognitive decline, even in the presence of a dementing illness. Cognitive reserve is also important for ADL, but only inasmuch as it protects against cognitive impairment. This contrasts with its effect on the behavioural and psychological symptoms of dementia where it is pre-morbid IQ rather than contemporaneous cognitive ability that is protective [25]. Our data are consistent with those of Pavlik and colleagues who followed up 478 AD patients over 3.2 years [26]. They investigated the effect of the American version of the NART on MMSE and ADAS-Cog scores together with the Clinical Dementia Rating Scale and found higher pre-morbid IQ, but not education, protected against decline on these global cognitive and functioning outcomes. By contrast a study from the Baylor College of Medicine [27] did not find such an effect on MMSE score decline, though the sample was smaller and thus may have been under-powered. Similarly Drachman and colleagues found few significant effects on the rate of decline in AD despite studying a wide range of predictor variables in a sample of just 42 patients [28]. On the other hand Mortimer and colleagues found a significant association between psychotic symptoms and the rate of cognitive decline in a sample of 65 patients [29]; we also reported a link between psychotic symptoms and cognitive status in our patients [25]. A Chicago study, which used a composite measure of pre-morbid reading ability, also failed to find a significant effect of this once other variables, including race, were adjusted for [30].

Though longitudinal studies have advantages for estimating effect sizes, they often suffer from the effects of attrition; Pavlik and colleagues also had over 90% attrition at the fifth wave of observation [26]. Indeed, there was deliberate selection involved in this study. Mixed linear models can adjust for such attrition to some degree because they include data from all participants, not just those who had observations made at every wave. Moreover, by using dummy variables indicating presence or absence at any wave, we could test for any attrition bias. There was none for NARTIQ; that is, people with lower pre-morbid IQs were no less likely to be assessed at week 26 (p = .13) or to be responders (p = .11). This finding, though, needs to be considered in the context of selection bias towards higher cognitive test scores for those patients who had a NARTIQ score. There were thus relatively fewer patients with very low cognitive tests scores in the sample analysed and thus it is possible that an attrition bias may have been found if all patients had had a NARTIQ and been included in the longitudinal study. Moreover, there was a selection bias for ADL, with people who were better at instrumental activities of daily living at baseline being more likely to be considered as responding to drug treatment. Hence, longitudinal effects of pre-morbid IQ on ADL may be less certain in those who presented with a lower level of self care.

In addition to attrition bias on ADL scores, the sample had an estimated pre-morbid IQ a little above average. However, given the mean age of the sample, this would be expected given the association between lower childhood IQ and shorter life expectancy [31]. The sample is also selected because of the nature of referral pathways to a tertiary clinic. Not only did the patients have to present to their general practitioner, but then had to be referred to a consultant and by the consultant to the memory treatment centre. This process generally took some time and thus patients who were deteriorating rapidly were unlikely to have reached the clinic. It may be, therefore, that any effects of cognitive reserve would have been less in patients who did not reach the clinic. Another limitation of the study is the range of cognitive tests. There were several cognitive domains that were not specifically tested that would be more or less influenced by cognitive reserve in AD. In addition, there were a number of explanatory factors that we did not take into account. Higher depression scores were associated with increased rate of decline in 102 Catholic clergy with AD [32]. However, low mood correlates with NARTIQ in AD patients [25], which was not taken into account, so that their findings could be explained by pre-morbid ability. One explanatory factor that was not open to us to investigate was the effect of race [30] because of the limited ethnic variation of Lothian residents. Nor did we take into account apolipoprotein E ε 4 status which has been implicated as a predictor of decline in AD [33]. These limitations indicate that further studies in other samples that can consider other explanatory variables would be useful.

## Conclusion

Pre-morbid IQ is an index of cognitive reserve. Our data support the hypothesis that cognitive reserve continues to influence cognition after AD has been diagnosed and thus, indirectly, has an impact on ADL. Not only does cognitive reserve influence the absolute level of performance, but it appears to ameliorate cognitive deterioration in AD patients, especially on tests with a high verbal content. The effect of cognitive reserve in AD is, however, fairly limited. Nevertheless, the degree of cognitive reserve requires consideration when making a diagnosis of AD and, to a lesser extent, when considering the likely future cognitive and ADL trajectories.

## Competing interests

The author(s) declares that they have no competing interests.

## Authors' contributions

JMS conceived the study and participated in its design, performed the statistical analyses and drafted the manuscript. JL helped to design the study, collected most of the data and helped draft the manuscript. Both authors read and approved the final manuscript.

## Pre-publication history

The pre-publication history for this paper can be accessed here:


